# Acute Exercise as an Intervention to Trigger Motor Performance and EEG Beta Activity in Older Adults

**DOI:** 10.1155/2018/4756785

**Published:** 2018-12-23

**Authors:** Lena Hübner, Ben Godde, Claudia Voelcker-Rehage

**Affiliations:** ^1^Professorship of Sports Psychology, Institute of Human Movement Science and Health, Chemnitz University of Technology, Thüringer Weg 11, 09126 Chemnitz, Germany; ^2^Psychology & Methods, Focus Area Diversity, Jacobs University Bremen gGmbH, Campus Ring 1, 28759 Bremen, Germany

## Abstract

Acute bouts of exercise have been shown to improve fine motor control performance and to facilitate motor memory consolidation processes in young adults. Exercise effects might be reflected in EEG task-related power (TRPow) decreases in the beta band (13–30 Hz) as an indicator of active motor processing. This study aimed to investigate those effects in healthy older adults. Thirty-eight participants (65–74 years of age) were assigned to an experimental (EG, acute exercise) or a control group (CG, rest). Fine motor control was assessed using a precision grip force modulation (FM) task. FM performance and EEG were measured at (1) baseline (immediately before acute exercise/rest), (2) during practice sessions immediately after, (3) 30 minutes, and (4) 24 hours (FM only) after exercise/rest. A marginal significant effect indicated that EG revealed more improvement in fine motor performance immediately after exercise than CG after resting. EG showed enhanced consolidation of short-term and long-term motor memory, whereas CG revealed only a tendency for short-term motor memory consolidation. Stronger TRPow decreases were revealed immediately after exercise in the contralateral frontal brain area as compared to the control condition. This finding indicates that acute exercise might enhance cortical activation and thus, improves fine motor control by enabling healthy older adults to better utilize existing frontal brain capacities during fine motor control tasks after exercise. Furthermore, acute exercise can act as a possible intervention to enhance motor memory consolidation in older adults.

## 1. Introduction

Fine motor control performance declines with increasing age [1], affecting activities of daily and professional life [2]. Although older adults are able to learn new and relearn motor skills [3], the consolidation of motor memory is diminished in older adulthood [4–7]. Acute bouts of cardiovascular exercise facilitate neuroplasticity in the primary motor cortex (M1) and enhance corticospinal excitability [8]. These effects are not specific to lower extremity motor areas and muscles engaged during exercise but also apparent in motor areas responsible for upper limbs, indicating that exercise has a generalized effect on M1 [9, 10]. Accordingly, several studies have investigated the effect of acute exercise on upper extremity visuomotor performance as well as acute exercise as a possible intervention to trigger motor consolidation processes in healthy young adults [11–18]. However, previous studies were inconsistent with respect to the time points of measurement, definition of motor performance/learning, and respective results, and were conducted only with young adults.

### 1.1. Effects of Acute Exercise on Motor Behavior

In the present study, we distinguished between (fine) *motor performance,* indicating a temporary status of motor behavior; *initial motor learning,* representing the very early phase of motor skill acquisition, and *motor memory,* characterized as a stable improvement of motor performance relative to baseline after a certain delay after practice [12, 19].

The influence of a bout of acute exercise on *motor performance* is usually assessed by performing a motor task immediately after an exercise session. Findings in studies with young adults are inconsistent: some indicate better fine motor control performance immediately after moderate intensity exercise than after rest [11], whereas others do not report such effects after moderate [15, 16] or high-intensity exercise [14].

The effect of acute exercise on *initial motor learning* can be measured by practicing the motor task immediately after exercise. Two studies report enhanced learning after moderate intensity exercise in young adults [11, 16], but others did not find altered motor learning behavior after moderate [15, 20] or high-intensity exercise [14, 18] in upper limb tasks.

Consolidation of *motor memory* refers to the transformation from a fragile to a stable memory trace evolving during online and offline processes after motor practice [21–23]. Memory consolidation processes can be distinguished with respect to different time scales, such as *short-term*, i.e., seconds to hours, and *long-term memory*, i.e., hours to months [24]. Accordingly, motor learning and acute exercise literature have assessed short-term motor memory up to one hour after practice/exercise and long-term motor memory 24 hours or seven days after practice/exercise [12, 25]. In healthy young adults, short-term motor memory in upper limb motor tasks seemed unaffected by acute exercise (one hour after exercise: [12, 14]). In contrast, participants revealed improved upper limb performance compared to resting control groups 24 hours and seven days [12, 14, 17, 18] after a bout of high-intensity exercise as well as 24 hours and seven days after low-intensity exercise [18]. This indicates that exercise triggers long-term motor consolidation processes, probably mediated by enhanced levels of norepinephrine, nerve growth factors, or metabolic signaling [14].

However, the influence of a bout of acute exercise on *motor performance*, *initial motor learning,* or *motor memory* in upper extremity fine motor tasks has not yet been studied in healthy older adults [26]. Several studies have revealed that acute exercise benefits cognitive performance in older adults immediately after moderate cardiovascular exercise [27–29], which was explained by higher arousal, improved information processing, and attention [28, 30]. As older adults require enhanced cognitive resources during motor performance and initial motor learning [31–33], and as motor consolidation processes are diminished in older adults [4–7], acute exercise might be an appropriate intervention to facilitate motor performance, initial motor learning, and motor memory consolidation in this age group.

### 1.2. Effects of Acute Exercise on Electrophysiological Data

Bilateral pre- and postcentral sensorimotor brain areas are involved in fine motor control performance [34–37], initial learning processes [38, 39], and consolidation of motor memory [23]. Task-related power (TRPow) decreases in the beta frequency band (13–30 Hz) over sensorimotor areas as obtained with electroencephalography (EEG) are discussed to be indicative of enhanced cortical activation and active processing of motor tasks [40–42]. In young adults, such desynchronization of beta oscillations has been shown during visuomotor force-matching tasks [41, 43, 44] and seems to reflect the efficiency of online and feedback processing of the motor system [45]. Furthermore, practicing a motor task led to weaker beta TRPow decreases not only in sensorimotor but also in frontal cortical areas in young adults [46–48], probably indicating increased automaticity and therefore, reflecting initial motor learning processes. With regard to age-related differences, in older as compared to young adults, stronger beta TRPow decreases during the performance of a force modulation task were found [49]. Following these results and as frontal brain activity is of particular interest in aging research [50], we focused our analysis on frontal and sensorimotor cortical areas by analyzing the beta frequency band.

Cardiovascular exercises have a modulating effect on activity in sensorimotor areas. For example, activity in the M1 or the primary somatosensory cortex (S1) was increased immediately after cardiovascular exercises [51–53]. In most studies, the cortical EEG was measured after acute exercise in young [54–57] and older adults [58] at rest. A recent study by Dal Maso et al. [59] investigated event-related desynchronization (ERD) during a power grip force modulation task after a bout of high-intensity exercise in young healthy adults. They revealed weaker ERD (analogous to less TRPow decrease) over bilateral sensorimotor cortical areas during early motor memory consolidation 30 to 90 minutes after exercise as compared to rest [59].

The aim of this study was, first, to investigate whether a moderate intensity cardiovascular exercise session facilitates (1) *motor performance*, (2) *initial motor learning* immediately after exercise, (3) *short-term motor memory* (30 minutes after exercise), and *long-term motor memory* (24 hours after exercise) in older adults. This was examined using a force modulation (FM) task performed with a precision grip. While existing studies with similar motor tasks conducted with young adults revealed controversial findings, effects might be more explicit in older adults due to age-related changes in motor processing. Greater improvement in fine motor control was expected during the motor performance and initial motor learning following acute exercise (experimental group) compared to after rest (control group). Furthermore, we hypothesized that the experimental group would show enhanced short-term and long-term motor memory consolidation, whereas the control group would not.

Secondly, this study aimed at investigating whether an acute exercise session influences TRPow in the beta band over the frontal and sensorimotor cortex during the performance of an FM task at the measurement points after exercise termination. We refer to TRPow decreases as indicators of enhanced motor processing [41, 42] and hypothesized that TRPow decreases were stronger directly after exercise as compared to after the control condition. Throughout practice, we expected that the experimental group would learn more than the control group, which might be reflected in stronger declines of the TRPow decreases. Analyses of EEG beta power at a reference spectrum at rest (before/after FM performance) were performed to confirm that beta power values did not differ between groups over time. Finally, we examined whether changes in beta TRPow were associated with changes in motor performance.

## 2. Methods

### 2.1. Participants

Forty-one older adults between the ages of 65 and 74 (69.51 ± 2.97 years of age, 22 female) participated in this study. Participants were recruited by local newspapers and were screened for the following prerequisites using telephone interviews: (1) age between 65 and 74, (2) absence of neurological and cardiovascular diseases, (3) physically active lifestyle, i.e., moderate intensity exercise for at least 150 minutes per week (in accordance to the recommendations of the American College of Sports Medicine [60]), and (4) right-handedness. The Ethics Committee of the Faculty of Humanities of the Saarland University (4.3.13) approved the study protocol. All participants took part voluntarily and provided informed consent regarding the general study information, receiving EEG and cardiovascular fitness tests, and also provided consent from their personal physician to complete the cardiovascular fitness test. Participants received 35 € as monetary compensation.

To validate the oral information and complete the screening process, participants answered a questionnaire assessing demographic information, education level (years of education), subjective health status (“in general, how would you say your health is?”—5-point Likert scale from *poor* to *excellent*), and physical activity level (adapted version of [61]). Participants were screened for dementia using the Mini-Mental State Examination (MMSE) [62], inclusion criteria ≥ 27 [63]. Further, they conducted the Edinburgh handedness inventory [64] to confirm their right-handedness (score: 84.95 ± 24.57) as well as a questionnaire to assess subjective hand use [65] to control for the exertion of fine motor activities during daily life. To control for restrictions in fine motor control, clinical manual dexterity was measured using the Purdue Pegboard test (Purdue Pegboard test, model 32020, Lafayette Instruments, Lafayette, IN, USA) [66]. The mean number of pins was calculated out of three trials placed with the dominant right hand. In the Purdue Pegboard test, all participants scored within the normative values for the right hand [67].

After screening and exercise testing, participants were assigned to an experimental (EG) or a control group (CG). Participants were matched with respect to their gender, age, MMSE score, and cardiovascular fitness level (VO_2_-peak, cf. below). Three participants had to be excluded from data analysis: one participant was not able to perform the fine motor control task adequately (EG), one was regarded as left-handed (CG) [64], and one due to noise in the EEG signal (EG). Therefore, 38 participants between 65 and 74 (69.68 ± 3.04 years of age, 20 female) were included in further analysis (descriptive statistics see Table 1). In the final sample, groups did not differ with respect to gender, age, education, subjective health, subjective hand usage, MMSE score, Pegboard performance, maximum voluntary contraction (MVC, see MVC task), physical activity, or cardiovascular fitness level (see Table 1).

### 2.2. Measures

#### 2.2.1. Cardiovascular Fitness Test

Cardiovascular fitness was measured by spiroergometry (ZAN600, nSpire Health, Oberthulba, Germany) on a stationary bicycle (Lode Corival cpet, Groningen, the Netherlands) using a ramp protocol to determine peak oxygen consumption (VO_2_-peak). The protocol was adjusted according to the participant's gender and self-reported physical activity level to ensure an adequate physical load. For this purpose, the participants were asked how often they perform cardiovascular exercise per week immediately before testing. If participants performed cardiovascular exercise less than three hours per week, a ramp protocol with a progressively increasing load of 10 W/min, starting with 10 W, was chosen for female participants, and a load of 15 W/min, starting with 10 W, was used for male participants. If participants exercised three hours or more per week, a progressive protocol starting at 10 W and increasing load of 15 W/min were used for females, and male participants started at 20 W and increased the load by 20 W/min. All tests were supervised by an experienced sports scientist. Electrocardiography (ECG, recorded with a ten-lead ECG fully digital stress system; Kiss, GE Healthcare, Munich, Germany), breath-by-breath respiration, heart rate, and blood pressure were continuously monitored. All spiroergometry protocols started with a three-minute rest period, then had participants cycled until a respiratory exchange ratio of 1.05 was maintained for about 30 seconds, and were finished with a five-minute cool-down period. Tests were terminated by volitional exhaustion or the occurrence of risk factors (i.e., systolic blood pressure > 230/115 mmHg, heart rate approximately > [220 – age]). The values of the highest complete performance level (about ≥ 4-5 seconds) achieved by the participants were averaged and regarded as VO_2_-peak, expressed as VO_2_ l/min.

#### 2.2.2. Fine Motor Control Performance and Learning: Apparatus and Setup

**Figure 1:**
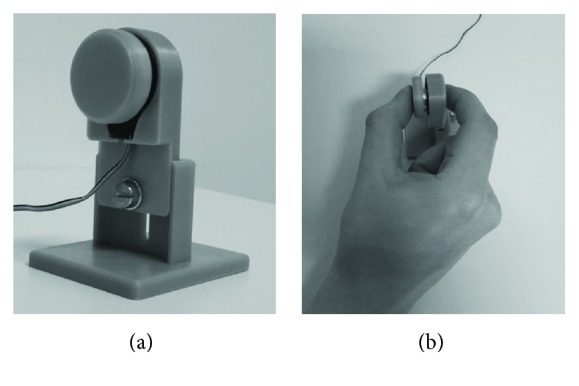
(a) Force transducer (FT). (b) Participant performs a precision grip.

Fine motor control was measured with a force transducer (FT, FX1901 OEM sensor, Variohm EuroSensor, Heidelberg, Germany). The sensor plate (diameter 25 mm) was encased with a plastic sheath (diameter 2.6 mm), which was located 45 to 71 mm above the tabletop on a 45 × 50 mm plastic pedestal (see Figure 1(a)). The FT was placed on a table in front of the participants in a comfortable position. Participants were seated about 60 cm in front of a 23.8-inch monitor (hardware resolution: 1920 × 1080 pixels). The monitor presented online visual feedback about the target (green) and applied force (yellow) on a black background. Target and applied force appeared on the right side of the screen and moved to the left. Target force appeared 200 ms before the applied force. Five seconds of force were presented continuously on the *x*-axis. For the maximum voluntary contraction (MVC) task, the *y*-axis was a fixed window from 0 to 100 N; for the force modulation (FM) task, the *y*-axis was set to 0 to 16 N. Force data were recorded at a sampling rate of 120 Hz and a resolution of 0.06 N with a customized LabVIEW program (LabVIEW 2015, National Instruments Austin, TX, USA). Participants had to pinch the FT with a precision grip, placing their thumb on the force sensor and the index finger on the plastic backside of the sensor (see Figure 1(b)). No participant had experience with this fine motor task. Participants received no feedback in terms of a quantified performance score.

#### 2.2.3. MVC Task

Participants' *maximum voluntary contraction* (*MVC*) was assessed by asking them to exert as much power as possible with their thumb and index finger on the FT (three trials of five seconds, ≥ 30 seconds rest between trials). The highest value out of the three trials was regarded as *MVC*.

#### 2.2.4. Force-Matching Task (FM Task)

**Figure 2:**
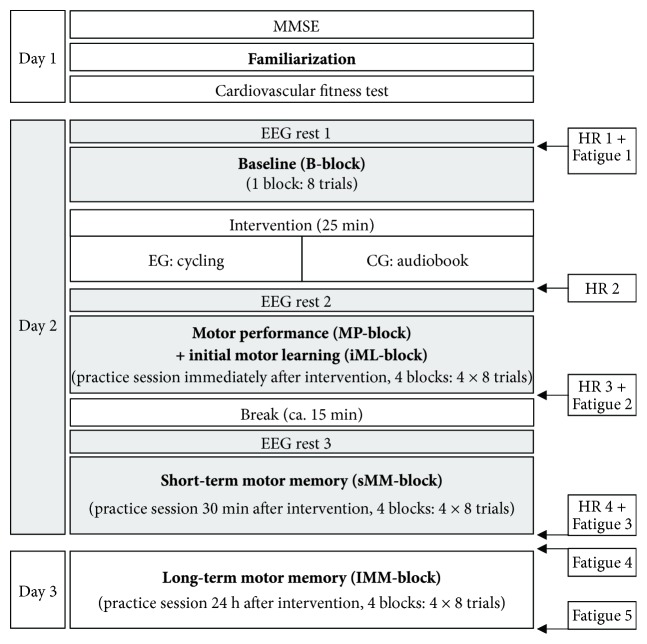
Schematic illustration of the study design. Italic text represents practice time points of the FM task. The grey boxes denote time points of EEG measurement. The white boxes on the right side represent measurement times of heart rate (HR) and subjective fatigue of the participants.

For an overview of the FM task procedure see Figure 2. First, *familiarization* for the FM task was performed. Participants were instructed to try out how the FT reacts during pinching with low forces without a target curve (one trial, length: 10 seconds). Then, participants had to apply force to match a target force as accurately as possible (part 1: constant target line at 4 N, three trials, 5 seconds each; part 2: regular sine-wave patterns between 1 N and 5 N, frequency of 0.4 Hz, three trials, 6.67 seconds each). For all FM task sessions, participants were again instructed to apply their own force to match the target curve as accurately as possible.

**Figure 3:**
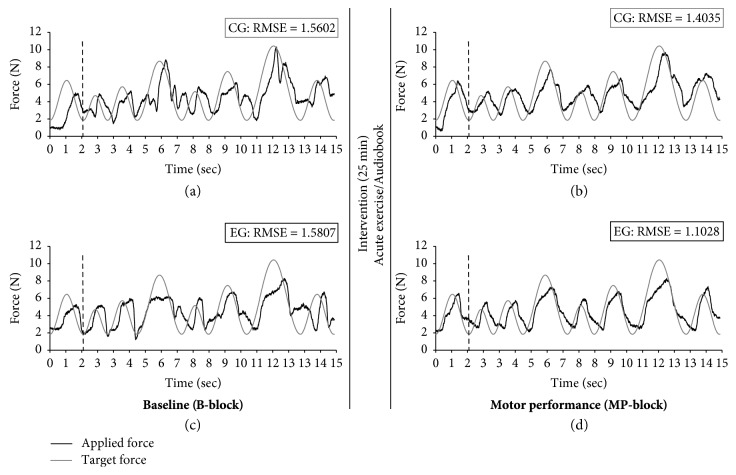
Example FM trials for one participant of each group (CG: a-b; EG: c-d) during *baseline* (*B-block*; a, c) and *motor performance* (*MP-block*; b, d). The grey target curves represent the irregular sine wave pattern. The black lines characterize the force applied by the participants. The black vertical dotted line symbolizes the start of data analyses.

The target curve of the actual FM task sessions consisted of an irregular sine-wave pattern of eight sine waves with the same minima (2 N) and varying maxima (5.1 N–11.3 N; see Figure 3). Sine-wave frequency (0.35 Hz–0.78 Hz) was adapted to the varying maxima so that tracking velocity was identical within each sine wave. The same sine-wave pattern was performed repetitively throughout the whole experiment. One trial had a length of 15 seconds. Trials were intermitted by a four-second intertrial break, during which a white fixation cross appeared on a black screen. During *baseline*, participants performed eight trials with the irregular sine wave. During the FM practice sessions, participants performed four blocks of eight trials immediately, 30 minutes, and 24 hours after intervention. The blocks were intermitted by breaks of approximately 30 seconds, during which participants were asked to relax their hands.

#### 2.2.5. Acute Exercise Session

The EG performed a moderate intensity exercise session by cycling on a stationary ergometer (Lode Corival cpet, Groningen, the Netherlands) for 25 minutes. The exercise started with a two-minute warm-up without Watt resistance, followed by 20 minutes at 60% of participants' maximum Watt performed during the cardiovascular fitness test (range: 54 W to 130 W, mean: 86.53 ± 25.17 W). The exercise session concluded with a three-minute cool-down without Watt resistance. Heart rate was monitored using a Polar A300 (Polar Electro Oy, Kempele, Finland) with an H7 heart rate sensor (Polar Electro Oy, Kempele, Finland).

#### 2.2.6. EEG Recording

Continuous EEG data were recorded with an active electrode system (actiCHamp, BrainProducts, Gilching, Germany) at a sampling rate of 500 Hz. Thirty-two electrodes were placed according to a modified 10–20 system [68] at the positions Fp1, Fp2, F7, F3, Fz, F4, F8, FC5, FC3, FC1, FC2, FC4, FC6, T7, C3, Cz, C4, T8, CP5, CP3, CP1, CP2, CP4, CP6, P7, P3, Pz, P4, P8, O1, Oz, and O2. The ground electrode was placed at position Fpz, and Fz was used as the reference electrode. Participants were instructed to sit comfortably on the chair and relax their facial muscles before EEG measurement started. EEG was recorded during the FM task as well as for 30 seconds immediately before and after all FM sessions on day 2 (i.e., baseline, practice immediately after, and 30 minutes after intervention) to calculate a reference spectrum (EEG rest). During these periods, participants were further instructed to sit calmly on their chair with both hands laying on the table and look at a white fixation cross on a black screen.

### 2.3. Procedure

Day 1: participants provided a written statement of consent from their physician for the cardiovascular fitness test and signed a declaration of consent for study participation. Then, they performed the MMSE, the FM *MVC*, and *familiarization*, followed by the cardiovascular fitness test. Day 2: the delay between days 1 and 2 was at least 48 hours (to ensure full recovery from the cardiovascular fitness test) and up to 14 days (mean: 5:27 ± 3:19 days). After EEG, caps were mounted and heart rate monitor was placed, participants started with the FM *baseline* measurement. Subsequently, EG performed the 25-minute acute exercise session in an adjoining room. CG stayed on the chair and listened to an audiobook (narrative short story) for 25 minutes. Participants performed the first practice session of the FM task immediately after the intervention (2 to 5 minutes). During the subsequent break (12 to 15 minutes), participants stayed on the chair and talked to one of the investigators. About thirty minutes (27 to 36 minutes) after the end of the intervention, participants performed another practice session. Participant's heart rate was recorded continuously during day 2 (see Figure 2; Table 2). EEG was measured only during day 2. Day 3: 24 hours after the intervention (range: 23.5 to 24.5 hours after the beginning of the first FM practice session on day 2) participants performed the last FM practice session. The order of testing days did not vary between participants. Fatigue of the performing right hand was controlled on a scale from zero (not at all fatigued) to ten (totally fatigued, see Table 2) at different time points on day 2 and day 3 (see Figure 2).

### 2.4. Data Analysis

#### 2.4.1. FM Task Performance

Data analysis of the FM task was processed in Matlab R2015b software (the MathWorks, Inc., Natick, Massachusetts, US). The first 211 data points (approximately 1.75 seconds, representing the first sine wave of every trial) were excluded from analysis to avoid variation due to differences in ramp time (cf. dotted vertical lines in Figure 3). Fine motor control performance was quantified using the root mean square error (RMSE) as a difference of the target and applied force. A mean of eight trials was calculated for *baseline* (= *B-block*), *motor performance* (= *MP-block*, i.e., first block of the practice session immediately after intervention), *initial motor learning* (= *iML-block*, i.e., block four of the practice session immediately after intervention), *short-term motor memory* (= *sMM-block*, i.e., first block of the practice session 30 minutes after intervention) and *long-term motor memory* (= *lMM-block*, i.e., first block of the practice session 24 hours after intervention). Outliers were presumed per trial across all participants as standardized *z*-scores greater than 3.29 or below −3.29 per trial (*n* = 11, in 7 different participants) and were replaced according to the last observation carried forward method [69].

#### 2.4.2. EEG Data

When not stated differently, the following preprocessing steps were performed identically for data from EEG rest and EEG during FM task. Offline EEG data processing was accomplished with the Brain Vision Analyzer software (Version 2.1, Brain Products GmbH, Gilching, Germany). A phase shift-free Butterworth infinite impulse response (IIR) filter was applied with a low cutoff at 1 Hz and a high cutoff at 70 Hz, with a slope of 48 db/Oct as well as a notch filter at 50 Hz to reduce line noise. Subsequently, a raw data inspection (criteria: gradient with a maximally allowed voltage step of 25 *μ*V, lowest allowed activity of 0.5 *μ*V) was performed. The electrode C4 revealed continuous artefacts for one participant. Accordingly, this channel was recalculated with a topographic interpolation for all measurement time points. To remove ocular artifacts, a semiautomatic ocular correction was conducted using an extended infomax independent component analysis (ICA), with the Fp1 electrode above the left eye detecting both vertical and horizontal ocular movements. Visual inspection confirmed that horizontal eye movements were detected and could be removed. Continuous EEG data during FM task were cut into segments from the beginning of the first trial of the force-tracking task to the end of the last trial of each session on day 2. Intertrial breaks and the first 1.75 seconds (corresponding to the curve of each trial, see Data Analysis FM task) were removed from the continuous EEG data during the task. All data during EEG rest were cut into 20-second segments (second 5 to 25 used for analysis). Continuous data were further separated into epochs of two seconds (overlapping segments of 150 ms), resulting in 7 epochs per FM trial and 10 epochs per EEG rest. Bad segments with obvious remaining artefacts were excluded based on visual inspection. In two participants, electrode CP3 and CP4 had to be excluded from further analyses for *EEG rest* 3, in one participant electrode CP4 had to be excluded for *EEG rest 1*.

After preprocessing, a fast Fourier transform (FFT) algorithm was applied (output was set to power measured in *μ*V^2^) using full spectrum and a Hanning window of 10% for each trial or each EEG rest, separately. Power spectra for the beta band (13–30 Hz) were calculated for six electrodes of interest, which are presumed to overlie the left (C3) and right (C4) M1, left (CP3) and right (CP4) S1, and the left (F3) and right (F4) frontal cortex. As operating the precision grip during the FM task required considerable activations of motor areas in both hemispheres [34, 36], power was calculated bilaterally. Power spectra were also calculated for each trial during FM task/EEG rest and each electrode separately. Subsequently, a mean power value for eight segments (corresponding to eight trials of the FM task, i.e., baseline/one block of practice) per electrode was calculated. Then, mean power values were log-transformed [42]. Finally, log-transformed task-related power (TR(logPow_x_)) was calculated by subtracting log-transformed beta power during EEG rest (*EEG rest 1*, *2*, and *3*, see Figure 2) from log-transformed beta power during FM task for each electrode separately: TR(logPow_x_) = log power task_x_–log power rest_x_ [42, 70], abbreviated TRPow in the following. For *B-block*, *EEG rest 1* was subtracted from the mean power value of *B-block*. For *MP-block*, *EEG rest 2* was subtracted from the mean power value of the first block directly after intervention. For *iML-block*, *EEG rest 2* from the mean power value of the fourth block directly after intervention. For *sMM-*block, *EEG rest 3* was subtracted from the mean power value of the first block 30 minutes after intervention (see Figure 2).

### 2.5. Statistical Analysis

All statistical analyses were conducted using SPSS for Windows, version 25 (IBM Corp., Armonk, NY, USA). If not stated differently, *p* values < .05 were regarded as significant, and *p* values < .10 as marginally significant (trend). The nominal alpha level was adjusted for particular analyses using Bonferroni adjustment (*α* = 1–(1–*α*)^1/m^); m = number of analyses/comparisons to control for multiple testing. Greenhouse-Geisser adjustment was reported in case the sphericity assumption was violated. Effect sizes were reported as partial eta squares (*η*_*p*_^2^). Demographic information (age, education, subjective health, and subjective hand usage), cognitive status (MMSE), MVC, cardiovascular fitness level (VO_2_-peak), and heart rate during day 2 were compared using analysis of variance (ANOVA) to assess any differences between EG and CG which may have affected motor performance (cf. Table 1).

#### 2.5.1. FM Task Performance

First, we performed a repeated measures analysis of variance (RM-ANOVA) with TIME (*B-block*, *MP-block*, *iML-block*, *sMM-block*, *lMM-block*) as within-subject factor and 2 GROUP (EG, CG) as between-subject factor to calculate the influence of the intervention (acute exercise or control condition) on FM performance and learning (Analysis 1). This was followed by three analyses to answer our a priori determined research questions, regardless whether the main effects of Analysis 1 were significant. A 2 TIME (*B-block*, *MP-block*) × 2 GROUP (EG, CG) RM-ANOVA (Analysis 1.1) was performed to investigate the influence of the acute exercise session on *MP-block* in the FM task. To examine the influence of acute exercise on *iML-block*, a 2 TIME (*B-block*, *iML-block*) × 2 GROUP (EG, CG) RM-ANOVA (Analysis 1.2) was conducted. Additionally, a 3 TIME (*iML-block*, *sMM-block*, *lMM-block*) × 2 GROUP (EG, CG) RM-ANOVA was performed to assess whether acute bouts of exercise facilitate *sMM*- and *lMM-block* (Analysis 1.3). The nominal alpha level was adjusted for Analyses 1.1–1.3 as well as for the post hoc *t*-tests of Analysis 1.3 using Bonferroni adjustment.

#### 2.5.2. EEG Data

A 4 TIME (*B-block*, *MP-block*, *iML-block*, *sMM-block*) × 2 HEMISPHERE (contralateral, ipsilateral) × 3 REGION (frontal, central, centro-parietal) × 2 GROUP (EG, CG) RM-ANOVA was calculated for TRPow (Analysis 2). Again, to answer a priori determined research questions and in correspondence to the behavioral FM task data, three further analyses were performed per electrode of interest. 2 TIME (*B-block*, *MP-block*) (Analysis 2.1) as well as 2 TIME (*B-block*, *iML-block*) × 2 GROUP (EG, CG) (Analysis 2.2) RM-ANOVAs for TRPow were calculated. Due to technical problems during *EEG rest 1* and *B-block* with the EEG, Analysis 2, 2.1, and 2.2 were conducted with *n* = 18 participants (instead of *n* = 21) for CG. As EEG was not assessed 24 hours after intervention, the last EEG analyses consisted of 2 TIME (*iML-block*, *sMM-block*) × 2 GROUP (EG, CG) RM-ANOVAs (Analysis 2.3).

Identical analyses were performed for *EEG rest 1*, *2*, and *3* to test whether acute exercise influenced beta power at rest: we conducted a 3 TIME (*EEG rest 1*, *EEG rest 2*, *EEG rest 3*) × 2 HEMISPHERE (contralateral, ipsilateral) × 3 REGION (frontal, central, centro-parietal) × 2 GROUP (EG, CG) RM-ANOVA (Analysis 3), followed by a 2 TIME (*EEG rest 1*, *EEG rest 2*) × 2 GROUP (EG, CG) (Analysis 3.1) as well as a 2 TIME (*EEG rest 2, EEG rest 3*) × 2 GROUP (EG, CG) (Analysis 3.3 RM-ANOVA. Note: *EEG rest 2* was used to calculate TRPow of *MP-block* as well as *iML-block.* For EEG at rest only a priori defined follow-up analyses per electrode were calculated. Again, the nominal alpha level was adjusted for Analyses 2.1-2.3, 3.1, and 3.3 as well as post hoc *t*-tests for Analysis 2 and 3 using Bonferroni adjustment to control for multiple testing.

FM and EEG analyses were controlled for a possible influence of participant age (see Table 1, marginal significant effect of age: *p* = .074). Age had no significant influence on FM or EEG statistics. Therefore, results were reported without the covariate age.

#### 2.5.3. Association between Behavioral Performance, EEG Data, and Load during Exercise

Subsequently, Pearson's correlation between FM performance (RMSE) and TRPow in analyses with significant TIME × GROUP interactions was performed to examine the association between behavioral performance and electrophysiological data. Moreover, Pearson's correlation between the Watt values performed during acute exercise and FM performance (RMSE) and TRPow were conducted to explore a possible association of exercise intensity and behavioral or electrophysiological data. Correlation analyses were controlled for multiple testing (Bonferroni adjustment).

## 3. Results

### 3.1. FM Task Performance

**Figure 4:**
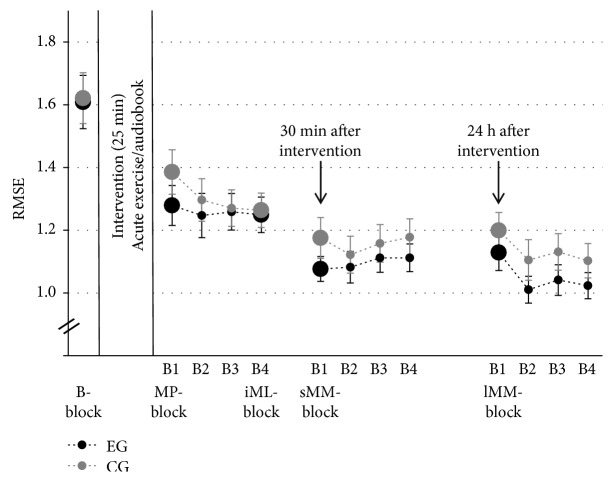
Means and SE of the FM task per group. Large circles indicate measurement time point included in statistical analysis: *B-block* = *baseline*, *MP-block* = *motor performance*, *iML-block* = *initial motor learning*, *sMM-block* = *short-term motor memory*, and *lMM-block* = *long-term motor memory*. Small circles indicate time points of practice blocks (B2-B4 = Block 2–4).

Figure 4 displays group means and standard errors (SE) of the FM task. Performance of EG and CG was similar at preintervention, differed immediately after intervention, and converged after the initial learning phase and motor memory consolidation. Analysis 1 confirmed a significant improvement for both groups from *B-block* to *lMM-block* (main effect of TIME: *F*(4, 36) = 73.99, *p* < .001, *η*_*p*_^2^ = .67), but no TIME × GROUP interaction (*F*(4, 36) = 1.00, *p* = .388, *η*_*p*_^2^ = .03) and no main effect of GROUP (*F*(1, 36) = 0.52, *p* = .475, *η*_*p*_^2^ = .01). Based on our a priori hypotheses, we further detail the results from three different analyses.

#### 3.1.1. Analysis 1.1

**Figure 5:**
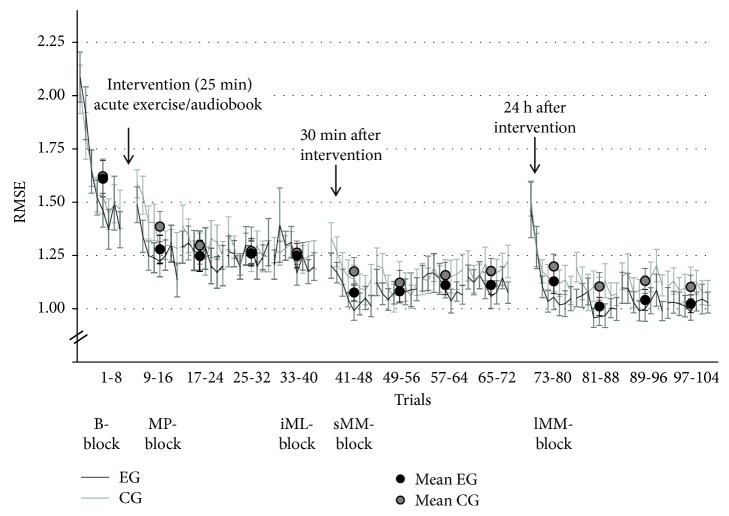
Mean and SE for all FM trials for EG and CG. The black (EG) and gray (CG) circles represent the means per block.

Effect of acute exercise on FM *motor performance (MP-block;* adjusted *α* = .017; see Table 3(a)). Performance of EG and CG was similar at *B-block* (preintervention) and improved in the first block of practice immediately after intervention (acute exercise or control condition: listening to an audiobook), with a more pronounced improvement in EG than CG. Figure 3 displays representative grip force profiles of one participant from CG and one from EG, and illustrates the different development from pre- to postintervention. More specifically, Figure 5 indicates that both groups improved similarly at *B-block*, but that EG learned more than CG at *MP-block*. We confirmed the development of CG and EG by a RM-ANOVA revealing a main effect of TIME on RMSE. TIME × GROUP interaction remained marginally significant after adjusting *α* level. A post hoc *t*-test further affirmed that EG and CG did not differ preintervention (*B-block: F*(1, 36) = 0.01, *p* = .918, *η*_*p*_^2^ = .01).

#### 3.1.2. Analysis 1.2

Effect of acute exercise on FM *initial motor learning* (*iML-block*; adjusted *α* = .017; see Table 3(b)). Exercise did not seem to foster motor learning during the first practice session after intervention, as FM performance in EG and CG improved similarly (see Figure 4). Analysis 1.2, comparing performance in *B-block* and performance in the last block of the first practice session (*iML-block*), resulted in a significant main effect of TIME, but no GROUP or TIME × GROUP interaction effect.

#### 3.1.3. Analysis 1.3

Effect of acute exercise on FM *motor memory* (*sMM-* & *lMM-block*; adjusted *α* = .017; see Table 3(c)). Motor memory was assessed 30 minutes (*sMM-block*) and 24 hours (*lMM-block*) after this initial phase of motor learning. Both groups revealed better *sMM-block* and *lMM-block* performance when compared to *iML-block* performance (main effect of TIME). Although TIME × GROUP interaction was not significant, we performed priori defined post hoc tests to specifically investigate the influence of exercise on short- and long-term motor memory consolidation. Post hoc *t*-tests between *iML-block*, *sMM-block*, and *lMM-block* per group (EG, CG) indicated that acute exercise significantly enhanced performance in *sMM-block* (*p* < .001) and in tendency in *lMM-block* (*p* = .027) compared to *iML-block*, whereas CG only revealed a tendency toward improvement in the *sMM-block* (*p* = .057), and no change in performance in the *lMM-block* (*p* = .321).

### 3.2. EEG Data

**Figure 6:**
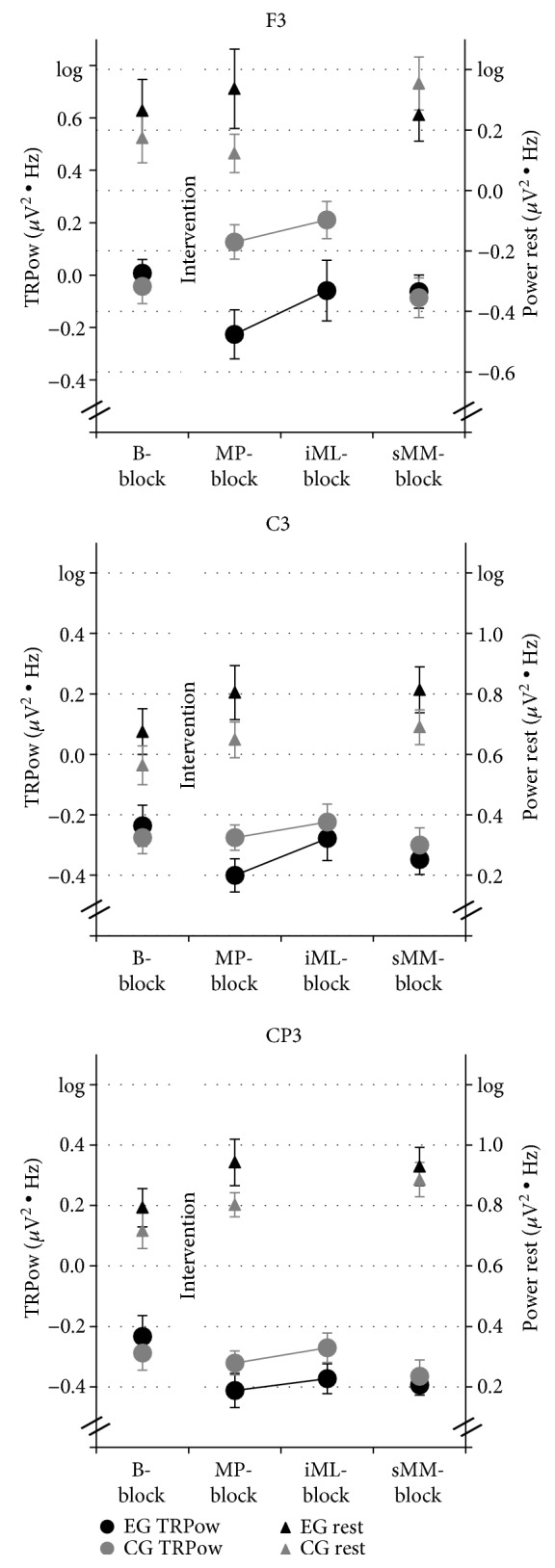
Means and SE of EEG TRPow (left *y*-axis) and EEG at rest (right *y*-axis) values in beta frequency band of the contralateral electrodes F3, C3, and CP3. Four measurement time points were included in the statistical analysis for TRPow (circles): *B-block = baseline, motor performance (MP-block) iML-block* = *initial motor learning*, *sMM-block* = *short-term motor memory*, and three for EEG at rest (triangles): *EEG rest 1*, *2*, and *3*.

Figure 6 shows TRPow group means and SE for different measurement time points (see Table 4 for all descriptive values for EEG beta power at rest and TRPow for EG and CG).

For the contralateral electrodes (F3, C3, CP3), TRPow of EG and CG was similar at *B-block*, different immediately after exercise, and coincided again after the initial learning phase. EG and CG did not differ for the ipsilateral electrodes (F4, C4, CP4).

#### 3.2.1. Analysis 2

Effect of acute exercise on TRPow. RM-ANOVA confirmed a significant effect of TIME (see Table 5(a) for all statistical results). As compared to *B-block*, the TRPow decrease was stronger in *MP-Block* directly after the intervention, weaker in *iML-block*, and even stronger during *sMM-block* (both compared to *MP-block*). A significant main effect of REGION revealed that TRPow decreases were stronger at centro-parietal and central than at frontal electrodes (centro-parietal/central vs. frontal: both *p* < .001, centro-parietal vs. central: *p* = .156). Furthermore, a significant TIME × HEMISPHERE interaction indicated that contralateral electrodes developed differently over time than ipsilateral electrodes. More specifically, TRPow decreases at contralateral compared to ipsilateral electrodes were weaker at *B-block* (*F*(1, 33) = 5.97, *p* = .020, *η*_*p*_^2^ = .15), and stronger at *MP-block* (*F*(1, 33) = 9.16, *p* = .005, *η*_*p*_^2^ = .22). A significant TIME × HEMISPHERE × GROUP interaction further indicated that the TIME × HEMISPHERE interaction stemmed mainly from regional differences in the EG group: TRPow decreases of contralateral compared to ipsilateral electrodes were weaker at *B-block* (*F*(1, 33) = 16.70, *p* < .001, *η*_*p*_^2^ = .34) and stronger at *MP-block* and *iML-block* (*F*(1, 33) = 12.08, *p* = .001, *η*_*p*_^2^ = .27; *F*(1, 33) = 16.21, *p* < .001, *η*_*p*_^2^ = .33, respectively). In contrast, post hoc *t*-tests did not reveal any significant difference in CG between contra- and ipsilateral electrodes at any time point (all *p* > .440). Finally, the TIME × GROUP interaction was marginally significant, indicating that the TRPow values of EG and CG tended to develop differently over the experiment.

#### 3.2.2. Analysis 3

Effect of acute exercise on EEG at rest (see Table 5(b)). The overarching analysis for EEG at rest revealed a significant TIME × HEMISPHERE × REGION × GROUP interaction. Generally, beta power increased from *EEG rest* 1 to *EEG rest 3* and from frontal over central to centro-parietal electrodes but differed with regard to electrode and hemisphere. Important to note, the follow-up TIME × GROUP interaction was not significant.

In line with our a priori hypotheses, EEG results were further analyzed using three distinct analyses.

#### 3.2.3. Analysis 2.1

**Figure 7:**
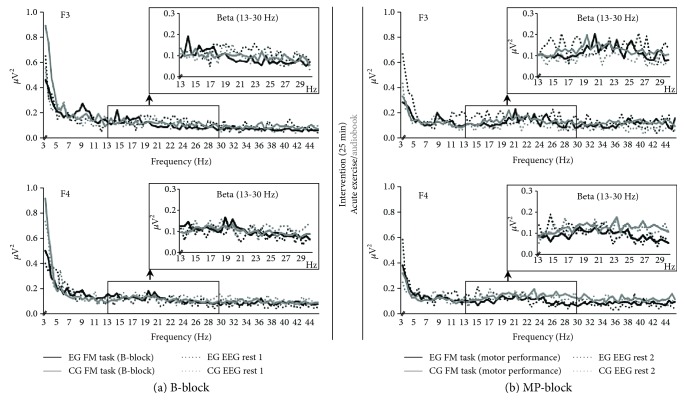
Means of the frequency (3–45 Hz) for EG (black line) and CG (grey line) for electrodes F3 (upper row) and F4 (lower row). The solid lines indicate data during FM task; dotted lines represent data at EEG rest. Data from FM trials during *baseline* (*B-block*) (a), *motor performance* (*MP-block*) (b), and *EEG rest 1* before intervention (a) and *EEG rest 2* after intervention (b) are illustrated. Inlets reveal the enlarged view of analyzed frequency band (beta: 13–30 Hz).

Effect of acute exercise on TRPow during FM *MP-block* (adjusted *α* = .017, see Table 6(a)).  Figure 7 illustrates group means of the power frequency spectra for EEG rest and during FM task separately for EG and CG for electrode F3 and F4. For electrode F3, EG and CG started at a similar beta power level during FM task (*B-block*), whereas EG revealed a tendency for more beta power at *EEG rest* (see also Figure 6, F3). After exercise during *MP-block*, beta power for EG was higher at EEG rest and lower during FM task compared to the *B-block*, reflecting a stronger TRPow decrease during *MP-block* than during *B-block* (see Figure 7 upper row). In contrast, participants in CG revealed a TRPow increase during *MP-block* (see Figure 7 upper row). This pattern was affirmed when comparing group means (Table 6(a): significant TIME × GROUP interaction) and calculating corresponding post hoc *t*-tests (EG: stronger TRPow decreases immediately after exercise (*F*(1, 33) = 12.53, *p* = .001, *η*_*p*_^2^ = .28); CG: marginally significant change from a small TRPow decrease to a TRPow increase immediately after intervention (*F*(1, 33) = 4.08, *p* = .051, *η*_*p*_^2^=.11). In contrast, such a group difference was not visible for electrode F4 (see Figure 7). Accordingly, statistical analyses confirmed no significant TIME × GROUP interaction for TRPow.

Furthermore, the main effects of TIME were significant for electrode CP3 and C4. For CP3, TRPow decreases became stronger from *B-block* to *MP-block*; TRPow decreases for C4 became weaker from *B-block* to *MP-block*.

#### 3.2.4. Analysis 2.2

Effect of acute exercise on TRPow during FM *iML-block* (adjusted *α* = .017; see Table 6(b)). TRPow decreases of contralateral electrodes at the end of the first practice session were mainly affected by the intervention (see Figure 6; significant TIME × GROUP interactions for contralateral F3 and marginally significant for CP3). Post hoc comparison for electrode F3 showed that TRPow decreases were significantly weaker during *iML-block* than during *B-block* in CG (*F*(1, 33) = 9.67, *p* = .004, *η*_*p*_^2^ = .23), whereas EG did not change significantly (*F*(1, 33) = 0.67, *p* = .418, *η*_*p*_^2^ = .02). In contrast, for electrode CP3, TRPow decreases became significantly stronger in EG (*F*(1, 33) = 7.98, *p* = .008, *η*_*p*_^2^ = .20), whereas CG did not change (*F*(1, 33) = 0.04, *p* = .841, *η*_*p*_^2^ < .01). As for *MP-block*, C4 revealed a TIME effect only, showing more TRPow decreases in EG and CG during *iML-block* after exercise compared to *B-block* in ipsilateral sensorimotor cortex.

#### 3.2.5. Analysis 2.3

Effect of acute exercise on TRPow during FM *sMM-block* (adjusted *α* = .017; see Table 6(c)). Thirty minutes after the intervention, TRPow values in EG and CG converged again, similar to *B-block* (see Figure 6). RM-ANOVAs comparing TRPow at *iML-block* and *sMM-block* confirmed a significant effect of TIME and TIME × GROUP interaction for electrode F3 (remained significant after adjusting alpha to *α* = .017). TRPow during *sMM-block* in CG was accompanied by stronger TRPow decreases in the contralateral frontal cortex compared to *iML-block* (*F*(1, 36) = 15.98, *p* < .001, *η*_*p*_^2^ = .31), whereas TRPow for EG remained stable (*F*(1, 36) < 0.01, *p* = .960, *η*_*p*_^2^ < .01). Again, for C4, we found a main effect of TIME, showing that TRPow decreases became stronger for both groups from *iML-block* to *sMM-block*.

#### 3.2.6. Analysis 3.1

Effect of acute exercise on *EEG rest 2* (adjusted *α* = .025; see Table 7(a)). Although EG revealed a trend for more beta power at electrode F3 for *EEG rest 1* (as noted above) and *rest 2* (see Figures 6 and 7), the main effect of GROUP and TIME × GROUP interaction were not significant. The main effect of TIME was significant for electrode CP3 and marginally significant for C3, indicating a general increase of beta power from *EEG rest 1* to *EEG rest 2*.

#### 3.2.7. Analysis 3.3

Effect of acute exercise on *EEG rest 3* (adjusted *α* = .025; see Table 7(b)). Beta power increased from *EEG rest 2* to *rest 3* at electrode F4 (TIME effect). For F3, the TIME × GROUP interaction was significant. Beta power increased for CG from *EEG rest 2* to *rest 3* (*F*(1, 33) = 7.33, *p* = .010, *η*_*p*_^2^ = .17) but remained stable for the EG (*F*(1, 33) = 0.84, *p* = .366, *η*_*p*_^2^ = .02).

### 3.3. Association between Motor Behavior, EEG Data, and Load during Exercise

A marginally significant correlation between FM task performance (RMSE) and EEG data (TRPow) was revealed for EG during *sMM-block* at electrode C4 (*r* = −.443, *p* = .075), pointing to a better FM performance with reduced TRPow decrease. However, after controlling for multiple testing, none of the correlation analyses reached statistical significance.

**Figure 8:**
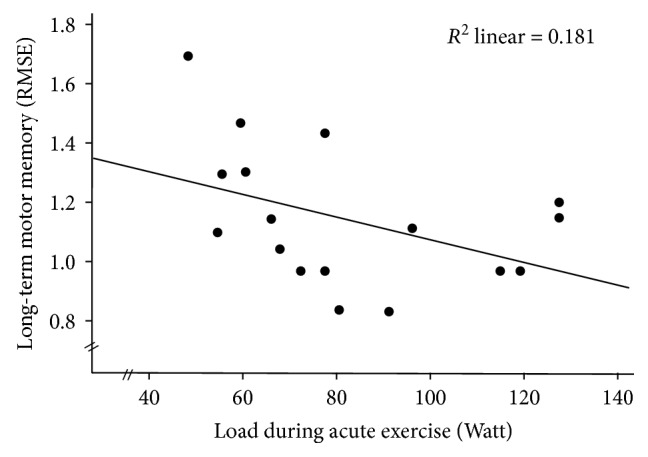
Scatterplot of the FM performance during long-term motor memory block (*lMM-block*) and the Watt load during acute exercise.

Correlation analyses between the Watt values performed during acute exercise and FM task performance (RMSE) indicated a marginally significant correlation (*r* = −.426, *p* = .088) for *lMM-block*: better FM performance 24 hours after exercise was associated with increasing Watt values (see Table 8 and Figure 8). After controlling for multiple testing, no correlation between Watt values performed during acute exercise and EEG TRPow revealed a significant association (see Table 9).

## 4. Discussion

This study examined the effect of an acute exercise session on (1) behavioral performance and learning in a precision grip force modulation (FM) task as well as on (2) the electrophysiological correlates of FM task performance and learning in healthy older adults. First, results revealed a marginally significant trend indicating that, as compared to *baseline*, participants of the experimental group (EG) improved their *motor performance* in the FM task immediately after exercise more than the control group (CG) after rest. Secondly, EG had steeper beta TRPow decreases (i.e., higher activity) than CG directly after exercise (measurement time point *motor performance*) at the contralateral frontal electrode, probably indicating that acute exercise facilitated motor compensation processes in the aged brain.

### 4.1. Effect of Acute Exercise on Motor Behavior

Although over the whole experiment both groups improved their performance with practice, our analyses revealed that acute exercise significantly influenced FM performance at particular time points.

#### 4.1.1. Motor Performance

In a sample of older adults, acute exercise facilitated *motor performance* in the FM task immediately after exercise in tendency more than a control condition. Similarly, Mierau et al. [71] reported more performance improvement in a motor adaptation paradigm immediately after exercise than after rest in young adults. The underlying mechanisms of superior motor performance immediately after exercise remain unknown. Findings from cognitive research revealed improved information processing indicated by electrophysiological markers (shorter latencies and larger amplitudes of the P3) immediately after exercise in older adults [29]. Skriver et al. [14] revealed that enhanced motor performance correlated with enhanced norepinephrine release and higher lactate level immediately after exercise in young adults. Furthermore, enhanced cerebral blood flow in the M1 during a finger tapping task [72] as well as an increase in the resting-state connectivity of the sensorimotor areas [73] in young adults immediately after exercise indicated better preconditions for motor task execution (see also Section 4.2).

#### 4.1.2. Initial Motor Learning

As especially, the initial phase of learning requires a high amount of cognitive resources [33], and acute exercise was found to improve cognitive performance in older adults [27–29], we examined whether acute exercise impacts this very early stage of learning. However, there was no superior effect of acute exercise on *initial motor learning* compared to a resting control condition in older adults. Thus, the positive influence of acute exercise on *motor performance* disappeared until the end of the practice session, revealing that the superiority of EG immediately after exercise was compensated by a higher performance gain of CG within the first FM practice session. We can only speculate about the underlying mechanism. One explanation might be that exercise itself already brought EG closer to their limits, whereas participants in CG made use of their capacity to improve during the practice session.

#### 4.1.3. Motor Memory Consolidation

There is a consensus that older adults, in general, reveal diminished long-term motor consolidation processes [4–7]. It was hypothesized that acute exercise could be a proper intervention to facilitate consolidation processes in older adults, because effects of acute exercise on *long-term motor memory* consolidation, but not *short-term motor memory* consolidation, have been shown in young adults [12, 14, 17]. However, such a positive effect of acute exercise could neither be confirmed on *short-term* nor *long-term motor memory* consolidation in our group of older adults. This seems to be in line with studies examining the effect of acute exercise on (not motor related) short- and long-term memory, exposing higher effects for young than for older adults [74]. Interestingly, the a priori post hoc tests revealed enhanced consolidation of *short-term* and *long-term motor memory* for EG after exercise, but only a tendency toward improvement of *short-term motor memory* for CG after rest. Thus, based on our results we cannot exclude that an acute exercise session might be a possible intervention to enhance motor consolidation in a FM task in older adults.

### 4.2. Effect of Acute Exercise on Electrophysiological Data

In addition to the FM data, the electrophysiological correlates of FM task performance were investigated. To this aim, beta TRPow decreases in electrodes supposed to lie over the frontal cortex, M1, and S1 were calculated as indicators of task-related cortical activation of the corresponding brain areas [41, 42].

In general, beta power at rest was highest over centro-parietal and lowest over frontal electrodes. Consistent with other studies [49, 75], also TRPow decreases were stronger at centro-parietal and central than at frontal electrodes. Although the exact origin of beta oscillations is unknown, the latter finding indicates a strong sensorimotor cortical activation during the motor task [76]. A trend for a TIME × GROUP interaction revealed that TRPow of EG and CG tended to develop differently throughout the experiment. For specific electrodes and time points, this was confirmed by our a priori defined analyses as discussed in the following.

#### 4.2.1. Motor Performance

The acute exercise session caused stronger TRPow decreases at electrode F3 (but not C3 or CP3), and therefore, higher activation of the contralateral frontal cortex during the FM practice session immediately after acute exercise. It has been shown that older as compared to young adults need to activate more frontal brain resources to successfully perform a motor task [50]. One prominent theory claims that this additional activation reflects compensation processes associated with maintained or enhanced motor performance [77]. Therefore, the increased contralateral frontal activity might indicate that acute exercise enables healthy older adults to better utilize existing frontal brain capacities during the FM task immediately after exercise. Our findings are also supported by functional near-infrared spectroscopy (fNIRS) studies, in which acute exercise as compared to rest led to compensatory frontal brain activity during the performance of a subsequent cognitive task in older adults [78, 79].

#### 4.2.2. Initial Motor Learning

It has been shown that practicing visuomotor force-matching tasks in general leads to an attenuation of beta TRPow decreases in frontal and sensorimotor cortical areas in young adults, interpreted as that task execution became less demanding due to increased automaticity [46, 48]. In line with these results, the CG in our study also revealed less contralateral frontal activation (less TRPow decrease) at the end of the first FM practice session compared to baseline. Interestingly, the contralateral frontal activity of the EG also decreased again in the course of the initial learning bock (after an initial increase of activity immediately after exercise, cf. 4.2.1), resulting in a nonsignificant effect from *baseline* to *initial motor learning*. As the cognitive load during the performance of a motor task decreases with practice [33], it might be that the initial strong compensatory contralateral frontal activity of the EG was not needed anymore to perform the FM task with ongoing practice. Furthermore, results indicate that acute exercise facilitated contralateral frontal beta activity directly after exercise but had no further effect on frontal beta activity during the learning course (similar to the behavioral FM data). In line with this finding, acute exercise led to a positive effect on cognitive performance only immediately after exercise in older adults, but not after a certain time delay [27].

#### 4.2.3. Motor Memory Consolidation

From the last block of the first FM practice session after intervention (i.e., *initial motor learning*) to the first block of the second FM practice session after a very short consolidation period (i.e., *short-term motor memory*), CG but not EG revealed a strong reduction of contralateral frontal cortical activity (i.e., weaker beta TRPow decreases). EG, in turn, did not change from *initial motor learning* to *short-term motor memory*, resulting in frontal TRPow decreases that did not differ significantly from the baseline level. Thus, the effect of the intervention (exercise or control condition) as well as the effect of practicing the FM task (first practice session) disappeared during the 15-minute break between FM practice sessions. Therefore, different to young adults [59], in older adults, efficient short-term motor memory consolidation was not reflected by weaker beta TRPow decreases. The different results might be related to the intensity of exercise, i.e., a moderate intensity exercise might not be strong enough to trigger motor memory consolidation in older adults or to a general age-related decline in memory consolidation after acute exercise [74].

Interestingly, the significant effects of acute exercise on EEG beta activity were restricted to the contralateral side; no influence was found for the ipsilateral brain areas. Although the FM task requires bilateral brain activity [34, 36], especially in older adults [49], acute exercise seemed to trigger the dominant contralateral brain side only.

A general increase of beta power at rest was revealed from *EEG rest 1* to *3.* Beta power at rest was interpreted as a correlate of processing of content-specific information [80, 81]. Therefore, one might assume that both groups increased processing throughout the FM practice on day 2 (regardless of the intervention). The lack of TIME × GROUP interactions for beta power at rest indicated that sweating did not systematically affect EEG data at rest.

### 4.3. Association between Motor Behavior, EEG Data, and Load during Exercise

After, correcting for multiple comparisons, no significant associations between FM task performance and TRPow during FM task were found. Thus, we might conclude that our results confirm earlier studies reporting no direct correlation between visuomotor performance, task-related beta power, and electrophysiological data [44, 82]. However, as the (marginally significant) correlation during short-term motor memory consolidation at electrode C4 for the experimental group was medium high, we might carefully interpret that as that good short-term motor memory consolidation came along with weaker TRPow decrease. This fits to our finding that TRPow decreases got weaker with practice. In contrast, a motor sequence learning study (without acute exercise) revealed that a high learning gain (i.e., a high decrease of reaction times) correlated with a high beta suppression after a short-term consolidation phase of ten minutes in young adults [83]. Diverging results might indicate different control strategies between the (key pressing) motor sequence task and our FM task and/or between age groups.

It was further investigated whether the individual exercise load was associated with FM task performance after exercise, as exercise loads varied highly within the EG (range: 54 W to 130 W). Although only marginally significant, there was a moderate association between exercise load and long-term motor memory consolidation, indicating better performance in the FM task 24 hours after acute exercise with higher exercise loads. The level of significance might be due to the small sample size of *n* = 17 and the high variation in the data. It has to be examined whether there is an objective criterion (i.e., exercise load in Watt) or relative criterion (exercise load as a percentage of maximum performance) that triggers these processes. Based on the correlational results, one could speculate that acute exercise needs to be performed at a certain exercise load to increase motor consolidation processes.

### 4.4. Limitations and Future Directions

To our knowledge, this study revealed for the first time that acute exercise facilitates fine motor control performance and learning as well as electrophysiological processing in healthy older adults. However, several factors might have influenced (or weakened) the effect of an acute exercise session on motor processes.

The aim of the *baseline* measurement was to assess a status quo of the initial fine motor performance of the participants. Participants in both groups revealed high improvements within these first eight trials of the FM task (see Figure 5). As the rate of improvement was nearly identical between the experimental and control groups, it rather represents a baseline familiarization than a baseline fine motor performance level. Based on the mean values, one could assume that the intervention time led to a short-term motor memory consolidation. However, considering the particular trials within the analyzed blocks during *baseline* and *motor performance*, we see that both groups started at a slightly lower performance level than the last trial of the baseline block. This contradicts the assumption of a general short-term consolidation due to the intervention break.

Although the intensity of exercise is discussed as an important factor in acute exercise research [84], no systematic association can be derived from studies using motor paradigms with young adults. That is, exercise intensity did not influence the consolidation of long-term motor memory systematically: better motor memory was found 24 hours after a high intensity [12, 14, 17, 18] and after a low-intensity exercise session [18], but, surprisingly, not after a moderate intensity exercise session [15, 16]. These heterogeneous results might have been influenced by the method of defining exercise intensity (% of VO_2_-peak vs. % of estimated age-related maximum heart rate vs. % of maximum Watt) or by the exercise type (cycling vs. running). In the current study, a moderate intensity exercise session was used, as this load could be transferred from laboratory-based acute exercise studies to the setting of rehabilitation, i.e., patients or persons not experienced with exercise [8]. High-intensity exercise did not seem appropriate, as it is performed by or recommended only for older persons with exercise experience [85, 86]. Nevertheless, exercise intensity might be a determining factor and should be systematically analyzed in future studies.

The order of acute exercise and practicing the motor task might also be an important factor. Roig et al. [12] found that practicing a motor task before acute exercise led to better long-term motor memory than practice after exercise. However, this finding was not supported by another study (using a motor adaptation paradigm), revealing that the order of practice and exercise did not influence the effect of exercise [87]. Thus, we decided to perform the practice sessions after exercise, as this design has been repeatedly shown to facilitate cognitive performance in older adults [27–29].

Furthermore, the physical activity/cardiovascular fitness level of the participants might mediate results. We generated a controlled sample in terms of the physical activity level, which could prevent generalizability to the general population of older adults. However, homogenizing the sample was necessary, as the physical activity level seems to mediate plasticity of the brain [88, 89] and the response to acute exercise sessions [90]. Further influencing factors might be the specific kind of upper extremity motor tasks (motor sequence learning: [91–94]; motor adaptation paradigms: [71, 87]), the kind of (cardiovascular) exercise [95], timing, or the duration of exercise. In sum, further studies with older adults are needed that systematically vary the potential influencing factors [26].

With respect to associated neural mechanisms, we restricted our analysis to the beta band (cf. introduction for argumentation). In addition, alpha oscillations (8–13 Hz) of the sensorimotor cortex were described as possible markers of sensorimotor processing [96, 97]. We did not find any acute exercise-related effects on EEG alpha power at rest or TRPow in our data (results not reported) and abstained from including it in the current report.

## 5. Conclusions

A moderate intensity acute exercise session tended to improve fine motor control performance immediately after exercise in a precision grip FM task in healthy older adults more than a resting control condition. Therefore, acute exercise might be a potential candidate to enhance motor performance in older adults. This could have important practical implications for the setting of rehabilitation: acute exercise could be implemented as a method to create successful experiences in fine motor control performance and therefore, to contribute in motivating older patients in the rehabilitation process. Further, the stronger contralateral frontal beta TRPow decreases immediately after the exercise session compared to after the control condition was interpreted as higher frontal brain activity [41, 42]. This higher beta activity might indicate enhanced compensation processes, implicating that acute exercise facilitates the ability to better use existing frontal brain capacities during fine motor control tasks.

## Figures and Tables

**Table 1:** Participant characteristics for EG and CG.

	EG (*n* = 17, 9 female)		CG (*n* = 21, 11 female)		*F-*statistics		
	*M*	*SD*	*M*	*SD*	*F*(1, 36)	*p*	*η*_*p*_^2^
Age	68.17	3.18	70.48	2.75	3.39	.074	.09
Education	16.38	2.17	15.76	1.99	0.84	.365	.02
Subj. health	4.12	0.60	4.10	0.63	0.01	.912	<.01
MMSE	28.88	0.86	28.52	0.98	1.40	.244	.04
Subj. hand usage	19.18	4.93	18.57	4.73	0.15	.698	<.01
Pegboard	12.35	1.13	12.19	1.72	0.11	.740	<.01
MVC	55.53	16.14	58.28	16.12	0.77	.387	.02
Physical activity	39.44	18.40	39.03	15.41	0.01	.940	<.01
Maximum Watt	142.82	42.68	143.00	35.73	< 0.01	.989	<.01
VO_2_-peak	1.95	0.59	1.85	0.53	0.25	.619	.01

Notes. EG = experimental group; CG = control group; age = age in years; education = years of education; subj. health = self-rated health status in a Likert scale from 1 (poor) to 5 (excellent); MMSE = sum score of the Mini-Mental State Examination; subj. hand usage = self-reported hand use (sum score of 9 items, 5-point scale); Pegboard = mean score of three trials with the right hand; MVC = maximum voluntary contraction of index finger and thumb, maximal value out of three trials; physical activity = kcal/kg^∗^wk; maximum Watt = maximum Watt performed during cardiovascular fitness test; VO_2_-peak = VO_2_-peak performed during cardiovascular fitness test in l/min.

**Table 2:** Descriptive results and *F*-statistics of heart rate and fatigue values at day 2 for EG and CG.

	EG (*n* = 17, 9 female)		CG (*n* = 21, 11 female)		*F-*statistics		
	*M*	*SD*	*M*	*SD*	*F*(1, 36)	*p*	*η*_*p*_^2^
HR 1	69.71	10.72	71.14	9.41	0.17	.680	.01
HR 2	99.92	11.20	n.a.	n.a.	—	—	—
HR 3	74.94	11.84	69.63	9.44	1.97	.171	.06
HR 4	69.46	11.65	68.07	7.62	0.17	.677	.01
Fatigue 1	0.66	0.83	0.81	1.03	0.24	.630	.01
Fatigue 2	2.35	1.46	2.18	1.93	0.10	.757	<.01
Fatigue 3	2.47	1.87	2.57	1.57	0.03	.858	<.01
Fatigue 4	0.71	1.21	0.86	0.91	0.19	.663	.01
Fatigue 5	1.25	1.44	1.60	1.57	0.48	.495	.01

Notes. EG = experimental group; CG = control group; HR = heart rate; Fatigue = subjective fatigue of the performing hand (scale from 0 to 10). HR 1 + fatigue 1: before baseline; HR 2: three minutes after acute exercise; HR 3 + fatigue 2: after practice session immediately after intervention; HR 4 + fatigue 3: after practice session 30 minutes after intervention; fatigue 4 + 5: before and after practice session 24 h after intervention.

**Table 3:** *F*-statistics for (a) Analysis 1.1, (b) Analysis 1.2, and (c) Analysis 1.3 for FM task performance (adjusted *α* = .017).

Analysis	*F*-statistics			
	*F*	*df*	*p*	*η*_*p*_^2^
(a) 1.1				
TIME	175.94	1	**<.001**	.83
GROUP	0.31	1	.579	.01
TIME × GROUP	4.91	1	.033	.12
(b) 1.2				
TIME	101.43	1	**<.001**	.74
GROUP	0.20	1	.888	<.01
TIME × GROUP	<0.01	1	.975	<.01
(c) 1.3				
TIME	12.62	2	**<.001**	.26
GROUP	0.67	1	.420	.02
TIME × GROUP	1.28	2	.284	.03

**Table 4:** Descriptive results for EEG beta power at rest and TRPow for EG and CG.

	EEG rest			EEG TRPow			
	*1*	*2*	*3*	*B-block*	*MP-block*	*iML-block*	*sMM-block*
F3							
EG	.26 ± .10	.34 ± .13	.25 ± .09	.01 ± .05	−.23 ± .09	−.06 ± .12	−.06 ± .06
CG	.17 ± .08	.12 ± .06	.35 ± .09	−.04 ± .07	.13 ± .07	.21 ± .07	−.09 ± .08
C3							
EG	.67 ± .08	.08 ± .09	.81 ± .08	−.24 ± .07	−.40 ± .06	−.28 ± .07	−.35 ± .05
CG	.56 ± .06	.65 ± .06	.69 ± .06	−.28 ± .05	−.28 ± .04	−.22 ± .06	−.30 ± .06
CP3							
EG	.79 ± .06	.94 ± .08	.93 ± .06	−.23 ± .07	−.41 ± .06	−.37 ± .05	−.39 ± .03
CG	.72 ± .06	.80 ± .04	.89 ± .06	−.29 ± .06	−.32 ± .04	−.27 ± .05	−.36 ± .05
F4							
EG	.13 ± .09	.13 ± .10	.26 ± .09	.10 ± .06	−.01 ± .08	.19 ± .11	−.13 ± .07
CG	.28 ± .09	.31 ± 07	.40 ± .07	.01 ± .06	.02 ± .06	.11 ± .06	−.11 ± .06
C4							
EG	.71 ± 09	.75 ± .09	.82 ± .08	−.57 ± .08	−.35 ± .06	−.26 ± .06	−.39 ± .06
CG	.53 ± .06	.66 ± .06	.70 ± 05	−.50 ± .04	−.24 ± .07	−.21 ± .06	−.30 ± .06
CP4							
EG	.83 ± .08	.86 ± 07	.90 ± .07	−.19 ± .08	−.23 ± .06	−.21 ± .06	−.23 ± .06
CG	.67 ± .06	.81 ± .05	.85 ± .05	−.11 ± .04	−.21 ± .05	−.15 ± .05	−.24 ± .05

Note. Mean ± SEM; EG = experimental group; CG = control group; B-block = baseline; MP-block = motor performance; iML-block = initial motor learning; sMM-block = short-term motor memory.

**Table 5:** *F*-statistics of (a) Analysis 2 (TRPow) and (b) Analysis 3 (EEG rest).

	*F*-statistics			
	*F*	*df*	*p*	*η*_*p*_^2^
(a) Analysis 2: TRPow				
TIME	5.03	3	**.009**	.13
HEMISPHERE	2.63	1	.114	.07
REGION	37.85	2	**<.001**	.53
GROUP	3.36	1	.076	.09
TIME × HEMISPHERE	4.65	3	**.017**	.12
TIME × REGION	2.03	6	.102	.06
TIME × GROUP	2.65	3	0.76	.07
HEMISPHERE × REGION	1.42	2	.250	.04
HEMISPHERE × GROUP	0.01	1	.920	<.01
REGION × GROUP	2.15	2	.131	.06
TIME × HEMISPHERE × GROUP	5.81	3	**.007**	.15
TIME × REGION × GROUP	0.76	6	.539	.02
TIME × HEMISPHERE × REGION	1.14	6	.343	.03
HEMISPHERE × REGION × GROUP	1.82	2	.169	.05
TIME × HEMISPHERE × REGION × GROUP	0.58	6	.669	.02
(b) Analysis 3: EEG rest				
TIME	7.07	2	**.002**	.18
HEMISPHERE	0.19	1	.665	.01
REGION	136.45	2	**<.001**	.81
GROUP	0.83	1	.370	.02
TIME × HEMISPHERE	0.48	2	.623	.01
TIME × REGION	1.13	4	.344	.03
TIME × GROUP	0.76	2	.460	.02
HEMISPHERE × REGION	0.52	2	.594	.02
HEMISPHERE × GROUP	6.18	1	**.018**	.16
REGION × GROUP	2.65	2	.098	.07
TIME × HEMISPHERE × GROUP	3.42	2	**.041**	.09
TIME × REGION × GROUP	0.70	4	.504	.02
TIME × HEMISPHERE × REGION	0.68	4	.535	.02
HEMISPHERE × REGION × GROUP	6.21	2	**.009**	.16
TIME × HEMISPHERE × REGION × GROUP	2.95	4	**.049**	.08

**Table 6:** *F*-statistics of (a) Analysis 2.1, (b) Analysis 2.2, and (c) Analysis 2.3 for EEG TRPow (adjusted *α* = .017).

	TIME				GROUP				TIME × GROUP			
	*F*	*df*	*p*	*η*_*p*_^2^	*F*	*df*	*p*	*η*_*p*_^2^	*F*	*df*	*p*	*η*_*p*_^2^
(a) Analysis 2.1												
F3	1.28	1	.266	.04	2.24	1	.144	.06	15.58	1	**<.001**	.32
C3	4.12	1	.051	.11	0.32	1	.577	.01	3.23	1	.081	.09
CP3	8.18	1	**.007**	.20	0.50	1	.825	<.01	3.59	1	.067	.10
F4	2.86	1	.100	.08	0.28	1	.603	.01	1.17	1	.287	.03
C4	28.75	1	**<.001**	.47	1.43	1	.240	.10	0.42	1	.524	.01
CP4	1.94	1	.173	.06	0.91	1	.348	.03	0.19	1	.670	.01
(b) Analysis 2.2												
F3	2.49	1	.124	.07	1.12	1	.297	.03	7.59	1	**.009**	.19
C3	<0.01	1	.995	<.01	<0.01	1	.971	<.01	1.01	1	.322	.03
CP3	3.55	1	.068	.10	0.07	1	.795	<.01	4.69	1	.038	.12
F4	1.73	1	.197	.05	1.24	1	.273	.04	0.07	1	.799	<.01
C4	71.83	1	**<.001**	.69	0.52	1	.476	.02	0.07	1	.799	<.01
CP4	0.04	1	.844	<.01	1.50	1	.229	.04	<0.01	1	.951	<.01
(c) Analysis 2.3												
F3	7.35	1	**.010**	.04	1.43	1	.240	.04	6.95	1	**.012**	.16
C3	2.83	1	.101	.07	0.49	1	.487	.01	0.01	1	.943	<.01
CP3	3.09	1	.087	.08	0.10	1	.753	<.01	1.27	1	.267	.03
F4	29.25	1	**<.001**	.45	0.15	1	.700	<.01	1.00	1	.336	.03
C4	9.36	1	**.004**	.21	0.68	1	.414	.02	0.36	1	.555	.01
CP4	2.42	1	.129	.06	0.08	1	.783	<.01	1.05	1	.312	.03

**Table 7:** *F*-statistics of (a) Analysis 3.1 and (b) Analysis 3.3 for EEG rest (adjusted *α* = .025).

	TIME				GROUP				TIME × GROUP			
	*F*	*df*	*p*	*η*_*p*_^2^	*F*	*df*	*p*	*η*_*p*_^2^	*F*	*df*	*p*	*η*_*p*_^2^
(a) Analysis 3.1												
F3	0.08	1	.780	<.01	2.14	1	.153	.06	2.29	1	.139	.07
C3	5.34	1	.027	.14	2.43	1	.128	.07	0.76	1	.388	.02
CP3	11.43	1	**.002**	.26	1.83	1	.185	.05	0.82	1	.372	.02
F4	0.03	1	.869	<.01	1.71	1	.200	.05	<0.01	1	.969	<.01
C4	3.94	1	.056	.11	2.07	1	.160	.06	0.65	1	.426	.02
CP4	2.34	1	.136	.07	2.34	1	.136	.07	0.65	1	.426	.02
(b) Analysis 3.3												
F3	1.28	1	.266	.03	21.55	1	.636	.01	6.21	1	**.017**	.15
C3	0.47	1	.449	.01	2.34	1	.135	.06	0.19	1	.665	.01
CP3	1.12	1	.298	.03	1.43	1	.239	.04	2.15	1	.151	.06
F4	6.37	1	**.016**	.15	0.16	1	.696	<.01	0.16	1	.696	<.01
C4	2.41	1	.130	.06	1.20	1	.281	.03	0.22	1	.644	.01
CP4	1.35	1	.253	.04	0.39	1	.535	.01	0.01	1	.910	<.01

**Table 8:** Correlations between the Watt load performed during exercise and FM performance (RMSE) after different time points of exercise (adjusted *α* = .013).

RMSE	Watt load during exercise	
	*r*	*p*
*MP-block*	−.291	.257
*iML-block*	−.300	.242
*sMM-block*	−.157	.547
*lMM-block*	−.426	.088

**Table 9:** Correlations between the Watt load performed during exercise and TRPow after different time points of exercise (adjusted *α* = .017).

TRPow	Watt load during exercise	
	*r*	*p*
*MP-block*	.217	.403
C3	.227	.381
CP3	.142	.585
C4	.334	.190
CP4	.378	.134
F3	−.069	.793
*iML-block*	.006	.981
C3	.336	.188
CP3	.195	.453
C4	.373	.140
CP4	.345	.175
F3	.112	.669
*sMM-block*	−.081	.758
C3	.171	.513
CP3	.559	.020
C4	.474	.054
CP4	.328	.198
F3	.377	.135

## Data Availability

The data used to support the findings of this study are available from the corresponding author upon request.
